# Dataset generated using hyperplexing and click chemistry to monitor temporal dynamics of newly synthesized macrophage secretome post infection by mycobacterial strains

**DOI:** 10.1016/j.dib.2016.08.055

**Published:** 2016-09-05

**Authors:** Ajay Kumar, Shilpa Jamwal, Mukul Kumar Midha, Baseerat Hamza, Suruchi Aggarwal, Amit Kumar Yadav, Kanury V.S. Rao

**Affiliations:** a International Centre for Genetic Engineering and Biotechnology, Aruna Asaf Ali Marg, New Delhi 110067, India; b Drug Discovery Research Center, Translational Health Science and Technology Institute (THSTI), NCR Biotech Science Cluster, 3rd Milestone, Faridabad-Gurgaon Expressway, Faridabad 121001, Haryana, India

**Keywords:** SILAC, BONCAT, Hyperplexing, Mtb infection, iTRAQ, Secretome, THP1 macrophage

## Abstract

Here we provide data for SILAC and iTRAQ based hyperplexing combined with BONCAT based click chemistry for selective enrichment of newly synthesized proteins secreted by THP1 macrophages at various time points after infection with four different strains of *Mycobacterium tuberculosis*. The macrophages were infected with H37Ra, H37Rv, BND433 and JAL2287 strains of *M. tuberculosis.* Newly-synthesized secreted host proteins were observed, starting from six hours post-infection till 26 h, at 4 h intervals. We have combined BONCAT with hyperplexing (18-plex), which blends SILAC and iTRAQ, for the first time. Two sets of triplex SILAC were used to encode the strains of *M. tuberculosis* - H37Ra & H37Rv in one and BND433 & JAL2287 in another with a control in each. BONCAT was used to enrich the secretome for newly synthesized proteins while 6-plex iTRAQ labeling was employed to quantify the temporal changes in the captured proteome. Each set of 18-plex was run in 4 MS replicates with two linear and two non-linear separation modes. This new variant of hyperplexing method, combining triplex SILAC with 6-plex iTRAQ, achieves 18-plex quantitation in a single MS run. Hyperplexing enables large scale spatio-temporal systems biology studies where large number of samples can be processed simultaneously and in quantitative manner. Data are available via ProteomeXchange with identifier ProteomeXchange: PXD004281.

**Specifications Table**

**Table:** 

Subject area	*Biology*
More specific subject area	*Proteomics*
Type of data	*Mass spectrometry raw files, Figure*
How data was acquired	*Mass spectroscopy, AB SCIEX 5600 triple-TOF*
Data format	*Raw & analyzed*
Experimental factors	*The secreted newly synthesized proteome of THP1 macrophages infected with different strains of Mycobacterium tuberculosis at six different time intervals*
Experimental features	*BONCAT labeling followed by SILAC, digested with Lys-C followed by trypsin. Peptide labeling with iTRAQ followed by sample pooling, separation through nano-LC and MS/MS analysis on AB SCIEX 5600 triple-TOF mass spectrometer.*
Data source location	*New Delhi, India*
Data accessibility	*Data is within this article and available in a Public repository via ProteomeXchange with identifier ProteomeXchange:*PXD004281

**Value of the data**

• This data characterizes the temporal physiological responses of macrophage secretome in response to four different strains of *Mycobacterium tuberculosis (H37Ra, H37Rv,* BND433 *and* JAL2287*).*
• Data could be used to understand the key regulators of host–pathogen interaction.
• This data can serve as a facile hyperplexing recipe for integrated BONCAT, SILAC and iTRAQ protocol for selective enrichment of low abundance secretome in a multiplexed manner.

## 1 Data

**Fig. 1:**
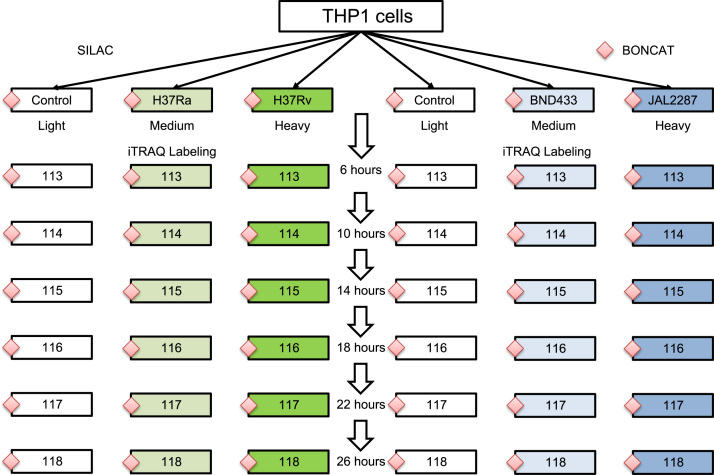
The schematic workflow showing the overview of the experiment.

The data contains 8 RAW file pairs (which contain 8 wiff and 8 corresponding wiff.scan files) from AB SCIEX 5600 tripleTOF instrument, each pair containing 18-plex multiplexing. Four files (two linear mode and two non-linear mode replicates) are from Set 1 and similarly the other four from Set 2. The overview of the experiment is shown in Fig. 1. The data also contains 8 zip archives containing MaxQuant [1] output and 8 quantification files from QuantWiz^IQ^ [2] output. Data is publicly available via ProteomeXchange with identifier ProteomeXchange: PXD004281 (http://proteomecentral.proteomexchange.org/cgi/GetDataset?ID=PXD004281).

## 2 Experimental design, materials and methods

### 2.1 Bacterial cell culture

All bacterial cultures were grown and maintained in Middlebrooke 7H9 broth (Difco) supplemented with 10% ADC (Becton Dickinson), 0.05% Tween 80 and 0.4% glycerol till the mid-log phase. Bacteria were harvested by centrifugation, washed with and re-suspended in RPMI 1640 (GIBCO). Cultures were prepared for infection by passing the bacterial suspension through 23, 26 and 30 gauge needles in tandem. Dispersed bacterial cultures were allowed to settle for 5 min and suspension from top was used for infecting THP1 cells. Bacteria were quantified by measuring absorbance at 600 nm wavelength (an optical density value of 0.6 corresponds to approximately 100 million bacteria). Source of studied mycobacterial strains i.e. H37Ra, H37Rv, BND433 and JAL2287 is as described elsewhere [3].

### 2.2 Host cell culture and infection with mycobacterial strains

THP-1, human monocytic cell line, was used for infection. The cells were differentiated using PMA for 48 h. Cells were either uninfected or infected with mycobacterial strains (mentioned above) at an MOI of 10 bacteria/cell, and samples were harvested at appropriate times post infection [3], [4].

### 2.3 Infection with mycobacteria and BONCAT

The cells at appropriate times were maintained either without any SILAC label (uninfected control) or with SILAC labels (mycobacteria infected cells). Azidohomoalanine was supplemented to uninfected and infected cells. Supernatants from each condition were collected at various time points post infection and spun at 1000 g to settle down any cellular debris. The samples collected were pooled and concentrated using 3KDa ultracentrifugal filters. The technique was recently referred to as BONLAC [5] to represent the combination of BONCAT with SILAC [6], [7] which has been demonstrated to be useful for studying selectively enriched newly synthesized proteome [8], [9].

### 2.4 Newly synthesized proteome enrichment and on-bead digestion

Newly synthesized proteins from concentrated media were enriched using Click-iT Protein Enrichment kit (Invitrogen C10416) as per manufacturer׳s instructions with slight modifications. For each reaction, the suggested reagent volumes were reduced to half. Sample and the catalyst solution were added to 100 µl of pre-washed agarose resin and kept at room temperature (RT) with constant shaking for 18–20 h incubation. The proteins were then reduced in the presence of SDS and dithiothreitol (DTT) (Bio-Rad) at 70 °C for 15 min, followed by alkylation in the presence of iodoacetamide (Bio-Rad). The resin was transferred into a spin column and washed with 10 ml of SDS buffer, 10 ml of 8 M urea in 100 mM Tris, pH8 and 20 ml of 20% acetonitrile (ACN). The resin was subsequently suspended in digestion buffer (100 mM Tris, pH8, 2 mM CaCl_2_ and 10% ACN) and transferred to a fresh tube. On-bead digestion was mediated by incubating samples with 0.5 µg LysC at 37 °C for 4 h followed by overnight incubation with 0.5 µg Trypsin (Promega). ACN was diluted to 2% with water and the diluted digested samples were acidified with formic acid (FA) followed by desalting with pre-equilibrated C18 reversed-phase columns (Waters). The pre-equilibration step included activation with 50% ACN/0.1% FA, followed by equilibration with 0.1% FA. After adding the sample, the column was washed twice with 0.1% FA. Desalted peptides were finally eluted in 50% ACN/0.1% FA, lyophilized and re-solubilized in ACN.

### 2.5 iTRAQ [10], [11] labeling

To enable simultaneous identification and quantification, multiplex iTRAQ reagent kit, from Sciex, was used to label peptides from 6 different biological samples, representing time points 6 h, 10 h, 14 h, 18 h, 22 h and 26 h per mycobacterial strain. Prior to labeling, all 6 iTRAQ reagents (113–118) were allowed to reach RT and supplemented with 50 µl isopropanol. Each time point sample was labeled with a single isobaric tag in the aforementioned order by transferring the contents from iTRAQ reagent vials to respective sample tubes. Samples, mixed with respective iTRAQ reagents, were subjected to 2 h incubation at RT. The tagged peptide mixtures from all sample tubes were pooled and dried by vacuum centrifugation followed by desalting through C-18 columns (Waters). The desalted samples were eluted in 40% and 60% ACN in 0.1% FA, respectively. Eluates were pooled and further cleaned by off-line strong cation exchange (SCX) chromatography using the SCX cartridge (Thermo Fischer Scientific). The tagged peptide mixtures were reconstituted in SCX low ionic strength buffer (5 mM ammonium formate, 30% ACN) pH 3 and loaded onto the cartridge and subsequently eluted with high cationic exchange buffer (500 mM ammonium formate, 30% ACN) pH 3. The samples were finally lyophilized prior to LC–MS/MS analysis. The combined MS^1^ and MS^2^ labeling, also called hyperplexing [12], enables 18-plex multiplexing in a single run of mass spectrometer.

### 2.6 NanoLC-mass spectrometry analysis—

All samples were analyzed by reverse-phase (RP) high-pressure liquid chromatography (HPLC) electrospray ionization coupled with tandem mass spectrometry (ESI-MS/MS) using a NanoLC-Ultra 1D plus (Eksigent; Dublin, CA) and nanoFlex cHiPLC system (Eksigent) directly connected to an 5600 Triple-TOF (AB SCIEX; Concord, Canada) mass spectrometer.

RP-HPLC was performed via an elute configuration using two Nano cHiPLC columns (Eksigent) in tandem to make a long column set up for better separation with good resolution; the analytical column (75 μm×15 cm) were manufacturer (Eksigent)-packed with 3 μm ChromXP C-18 (120 Å). Reverse-phase LC solvents included: mobile phase A: 2% ACN/98% 0.1% FA (v/v) in water, and mobile phase B: 98% ACN/2% 0.1% FA (v/v) in water. The auto-sampler was operated in full injection mode overfilling a 1 µl loop with 3 µl analyte for optimal sample delivery reproducibility. All samples were eluted in replicates from the analytical column at a flow rate of 300 nL/min by two different elution gradient modes: Linear and Step wise elution modes. Using linear gradient mode of 5% solvent B to 50% solvent B over duration of 300 min, separation was done. 90% solvent B for 20 min was used to regenerate the column, followed by re-equilibration with 5% solvent B for 40 min. During Non-Linear gradient mode, separation was done in step wise manner with 5% solvent B to 20% solvent B over duration of 180 min; 20% B to 30% B for 80 min; 30% B to 50% B for 30 min. The column was regenerated by washing with 90% solvent B for 20 min and re-equilibrated with 5% solvent B for 40 min. Auto-calibration of spectra after every 2 samples was programmed using dynamic LC–MS and MS/MS acquisitions of 50 fmol β-galactosidase.

Spectra were generated in positive-ion mode with a resolution of about 35,000 in high sensitivity mode. Samples were injected into the mass spectrometer using 10 μm SilicaTip electrospray PicoTip emitter (New Objective Cat. No. FS360-20-10-N-5-C7-CT). The data acquisition was carried out in DDA mode to obtain a high resolution TOF-MS scan in the mass-to-charge range 350–1250 m/z, followed by high sensitivity mode MS/MS scans of 15 ion candidates per cycle with activated rolling collision energy. The parent ions greater than 150 cps with a charge state between +2 to +5 were selected for MS/MS in a mass tolerance of 50 mDa and were present on a dynamic exclusion list for 10 s. The ion accumulation times for MS and MS/MS were set to 500 ms and 200 ms respectively.

### 2.7 Database search and relative quantification

The wiff files from AB SCIEX 5600 mass spectrometer were searched using MaxQuant (1.5.0.30) against Uniprot human database with cRAP sequences (www.thegpm.org/crap/) and their corresponding reversed sequences appended to it. The parameters for search were as follows - triple SILAC on Lysine and Arginine with mass of iTRAQ added to Lysine labels, fixed modifications used were- carbamidomethylation at cysteine & iTRAQ at N-term; variable modifications used were- methionine oxidation, BONCAT and deamidation at NQ residues. The results were filtered at 1% false discovery rate (FDR) [13] using MaxQuant. The wiff files were converted to mgf files using msconvert and iTRAQ labels were quantified using in-house developed QuantWiz^IQ^ tool. Using MaxQuant and in-house developed tools and integration pipelines, we have mapped the temporal and strain specific dynamics of newly synthesized proteins in host.
